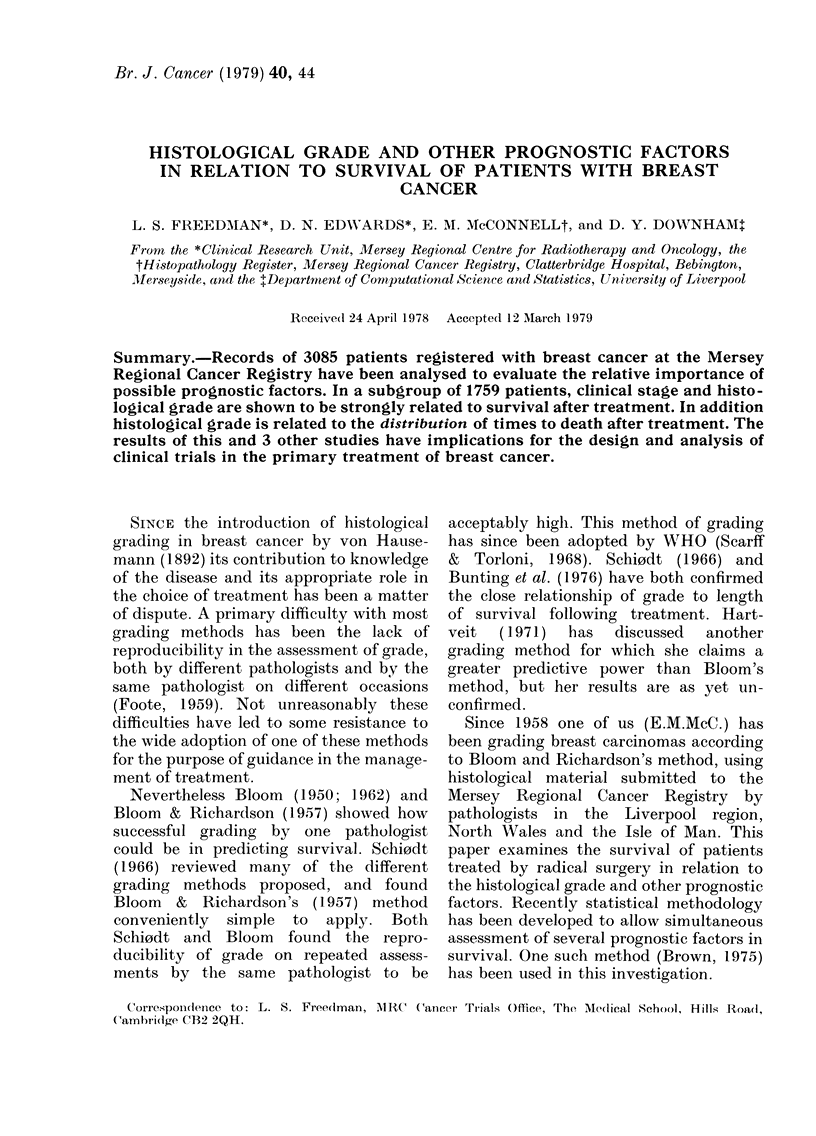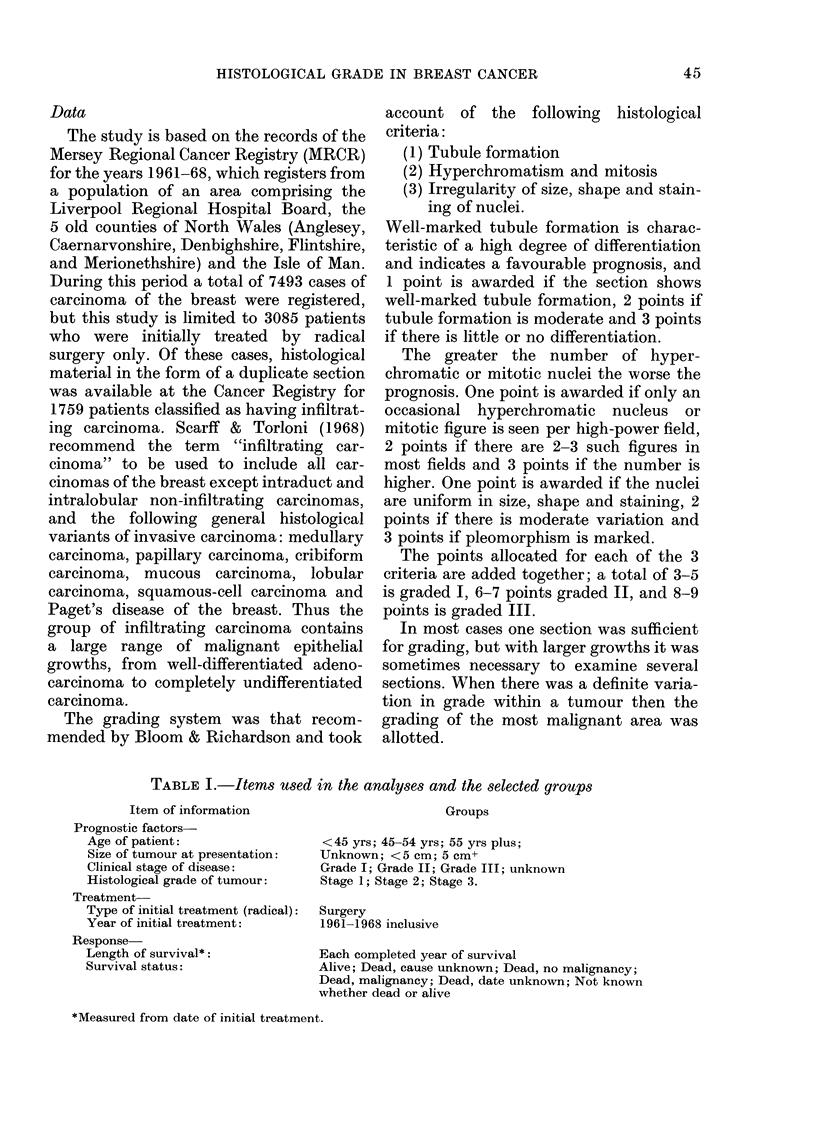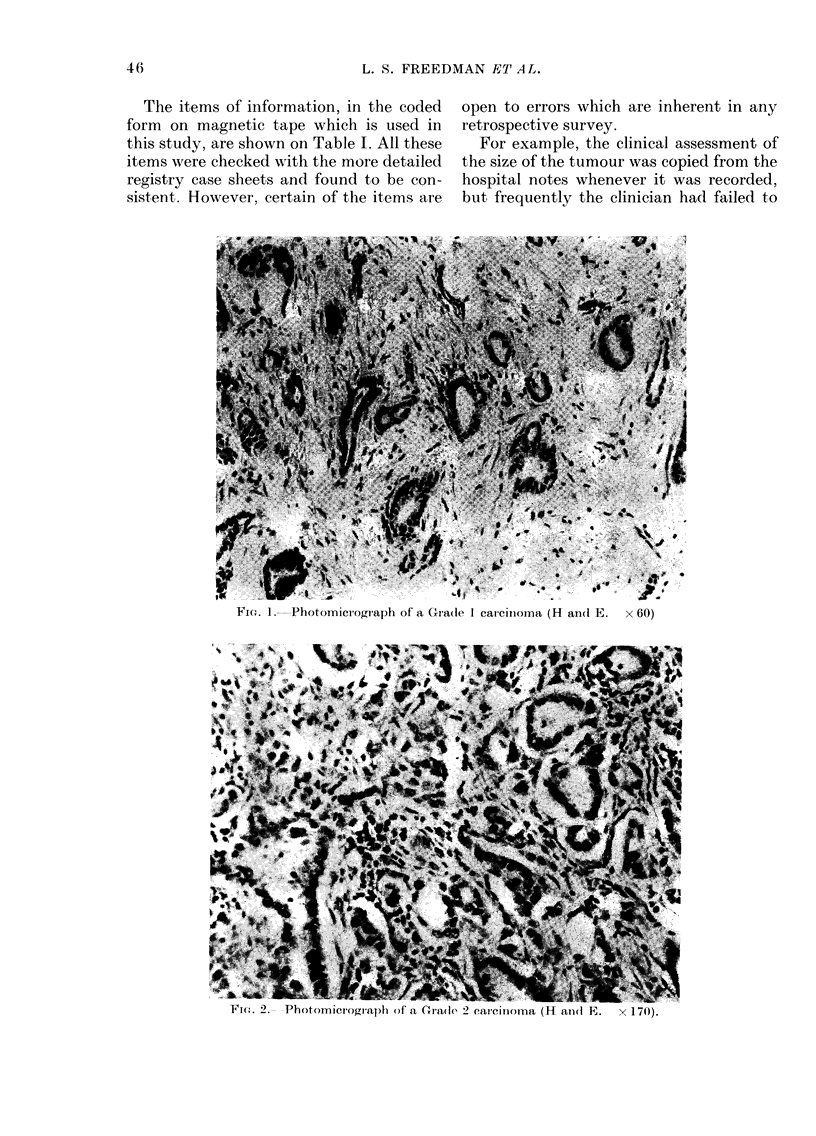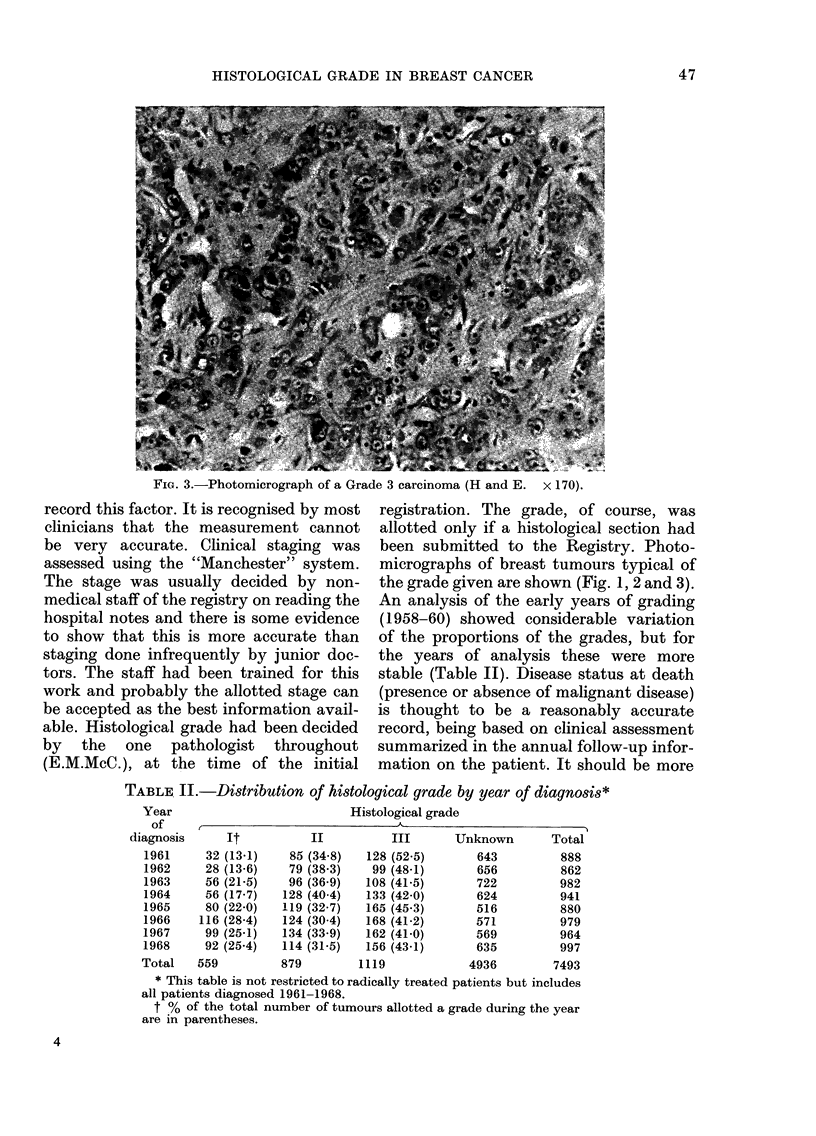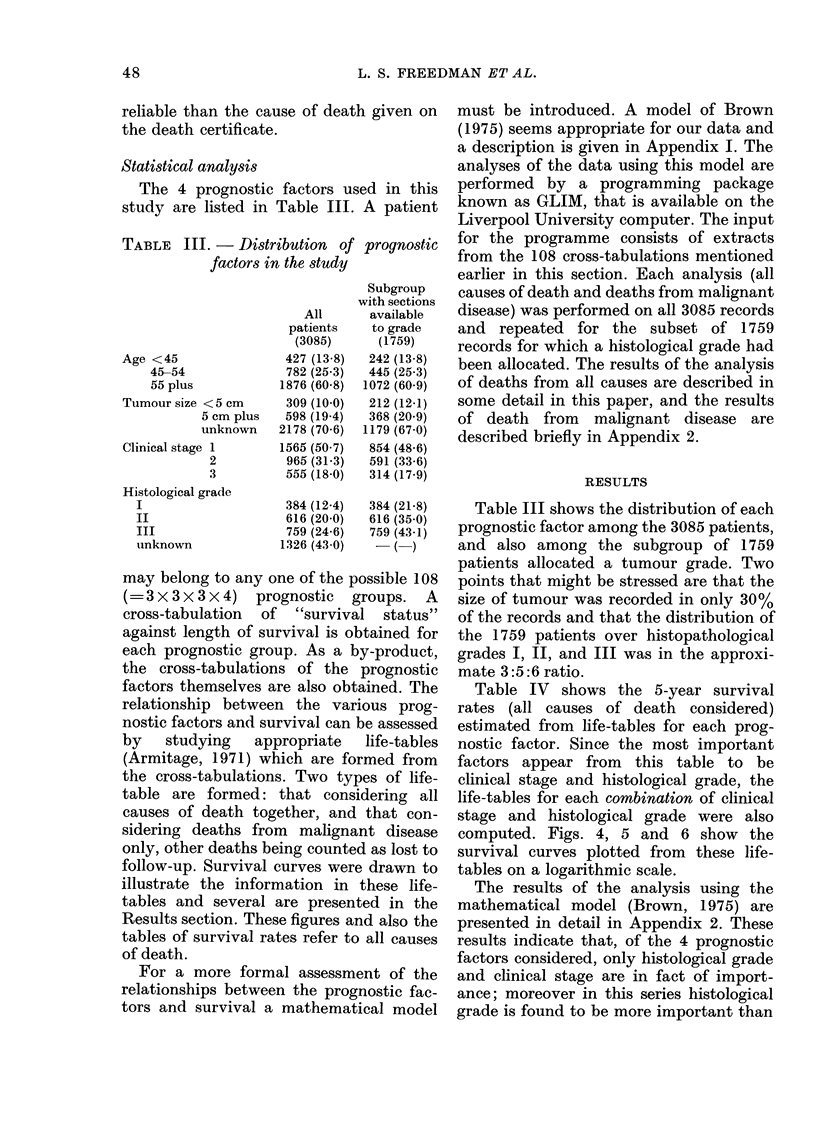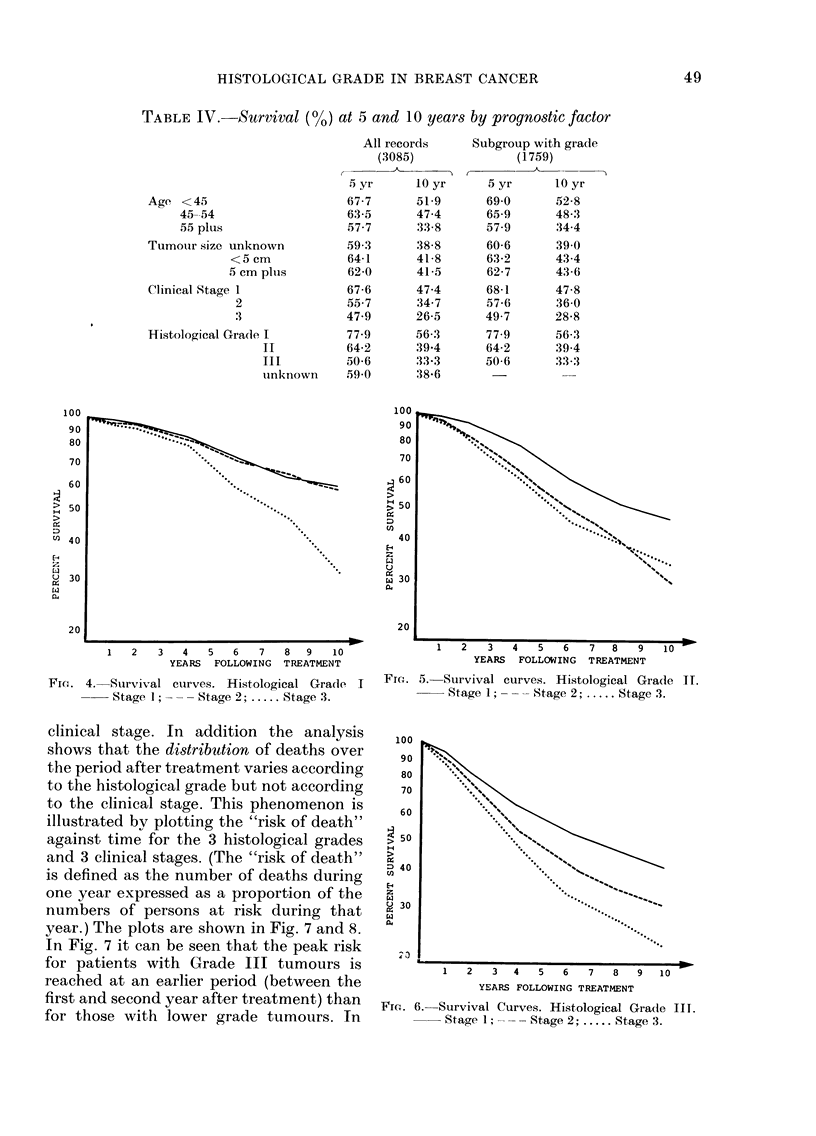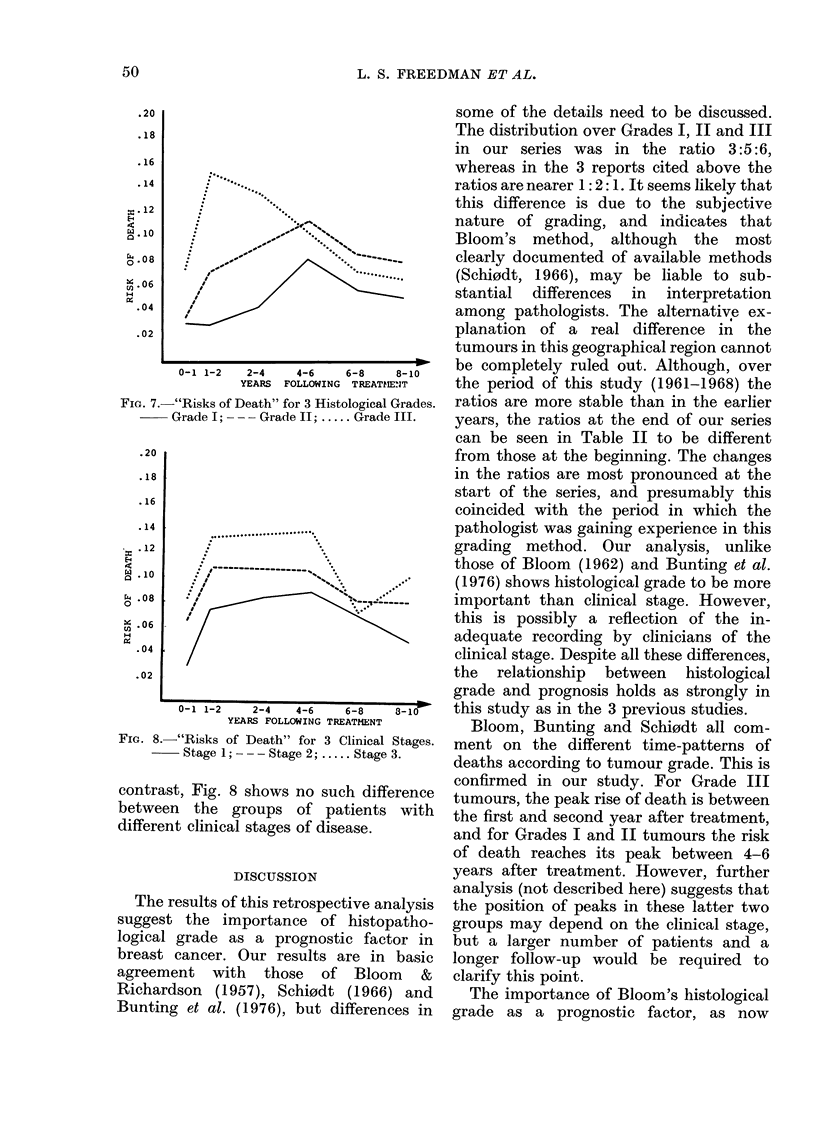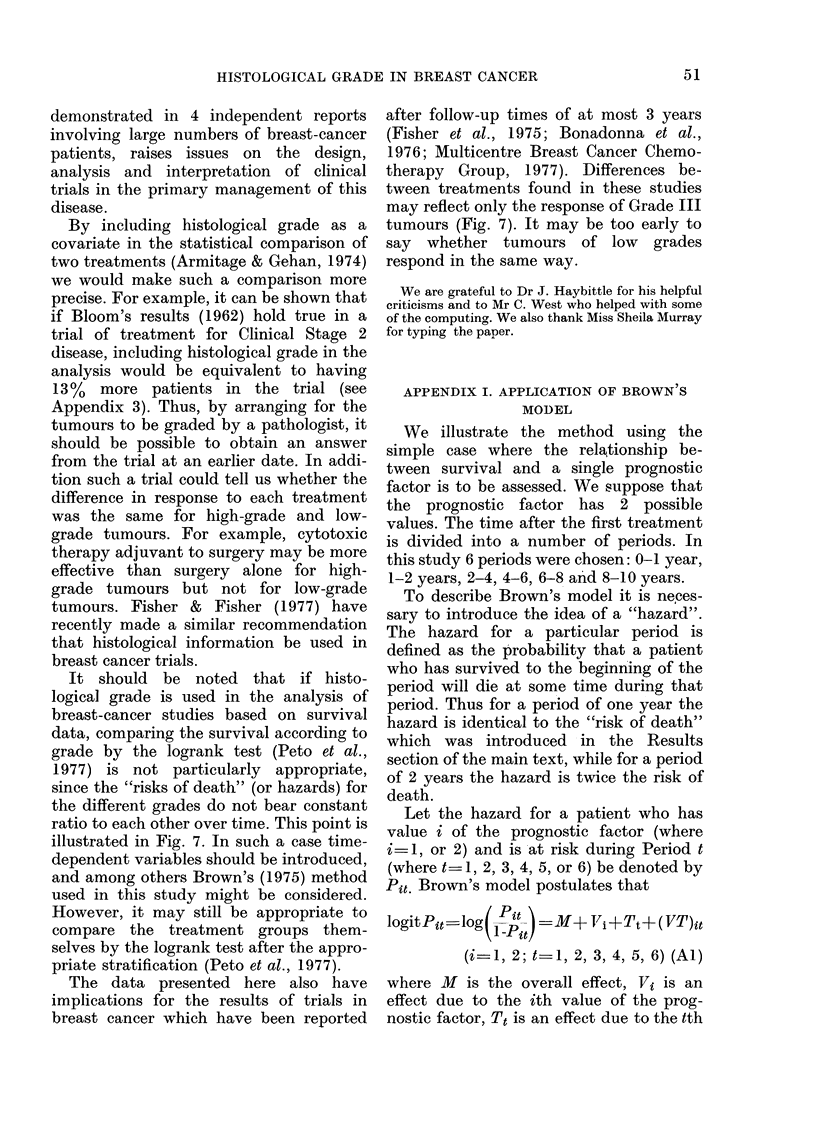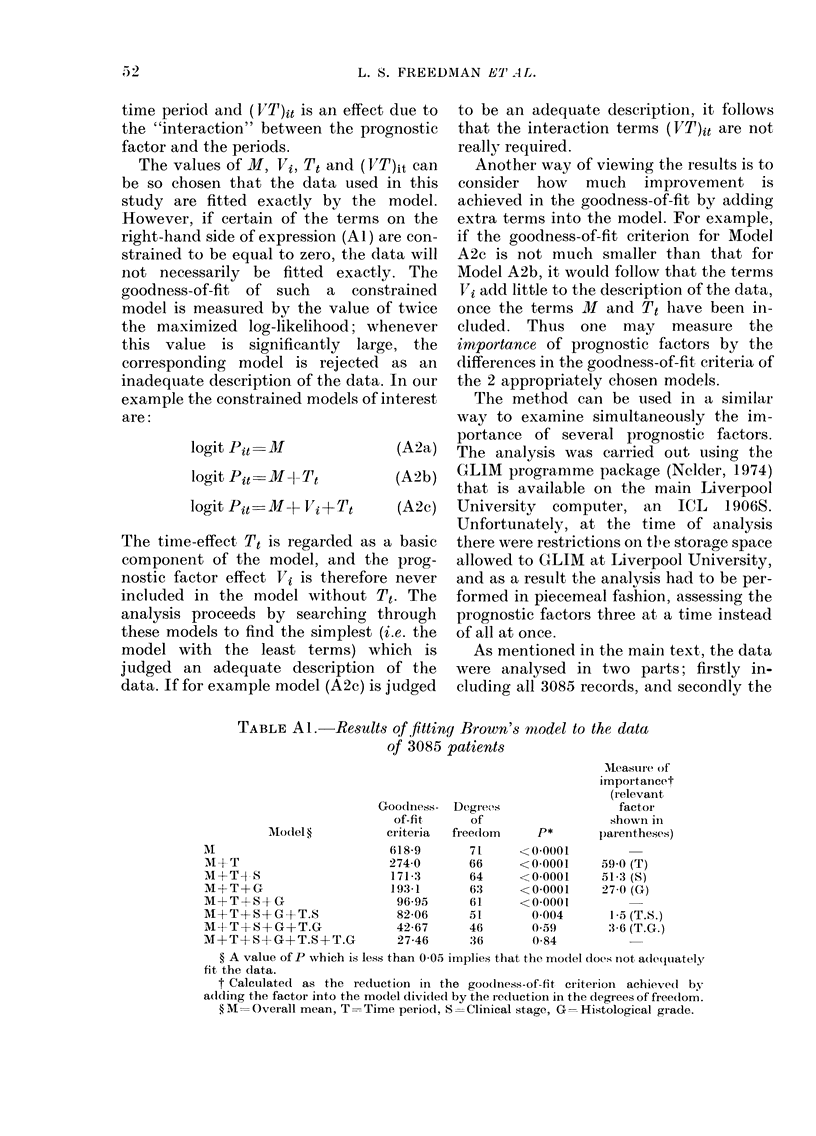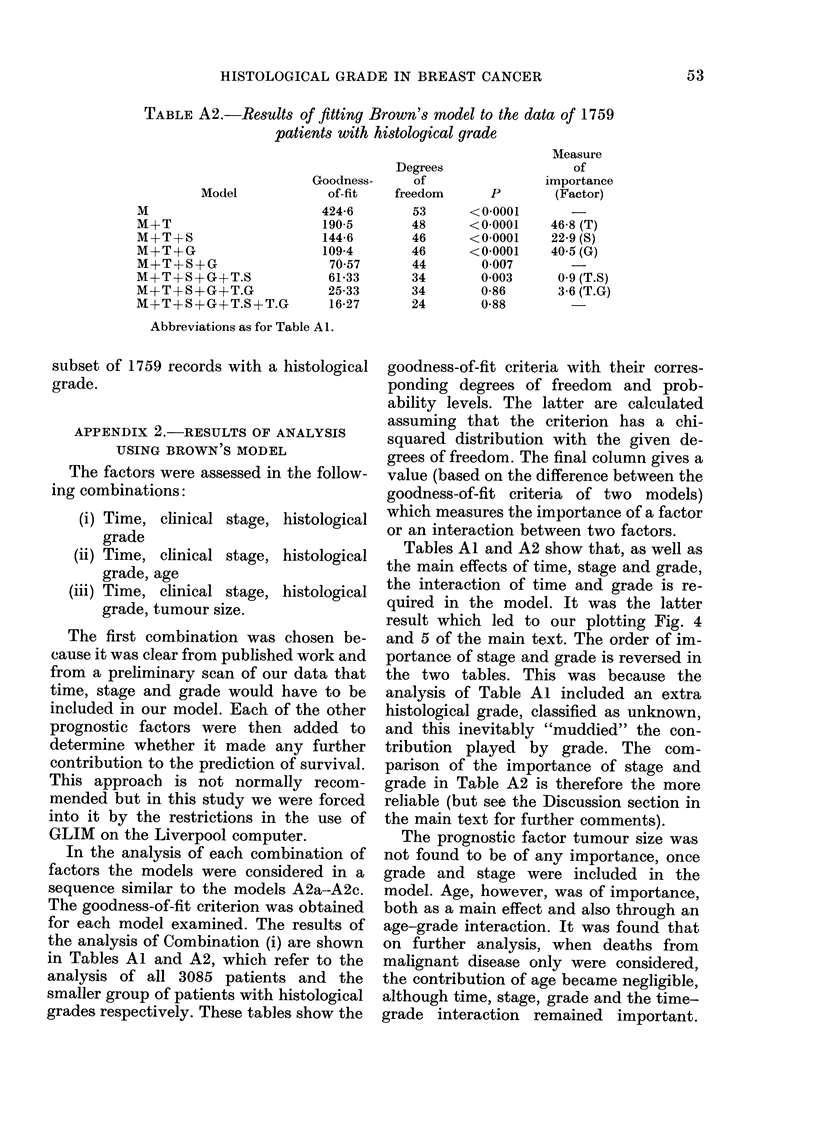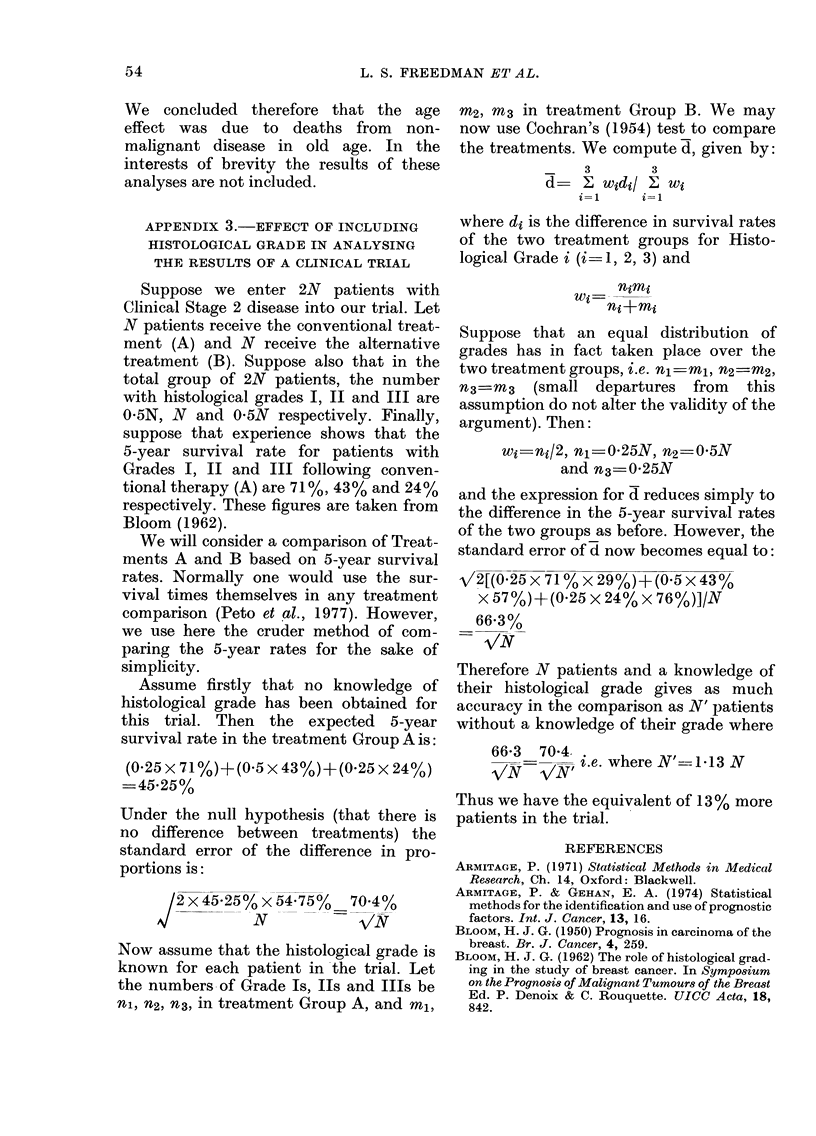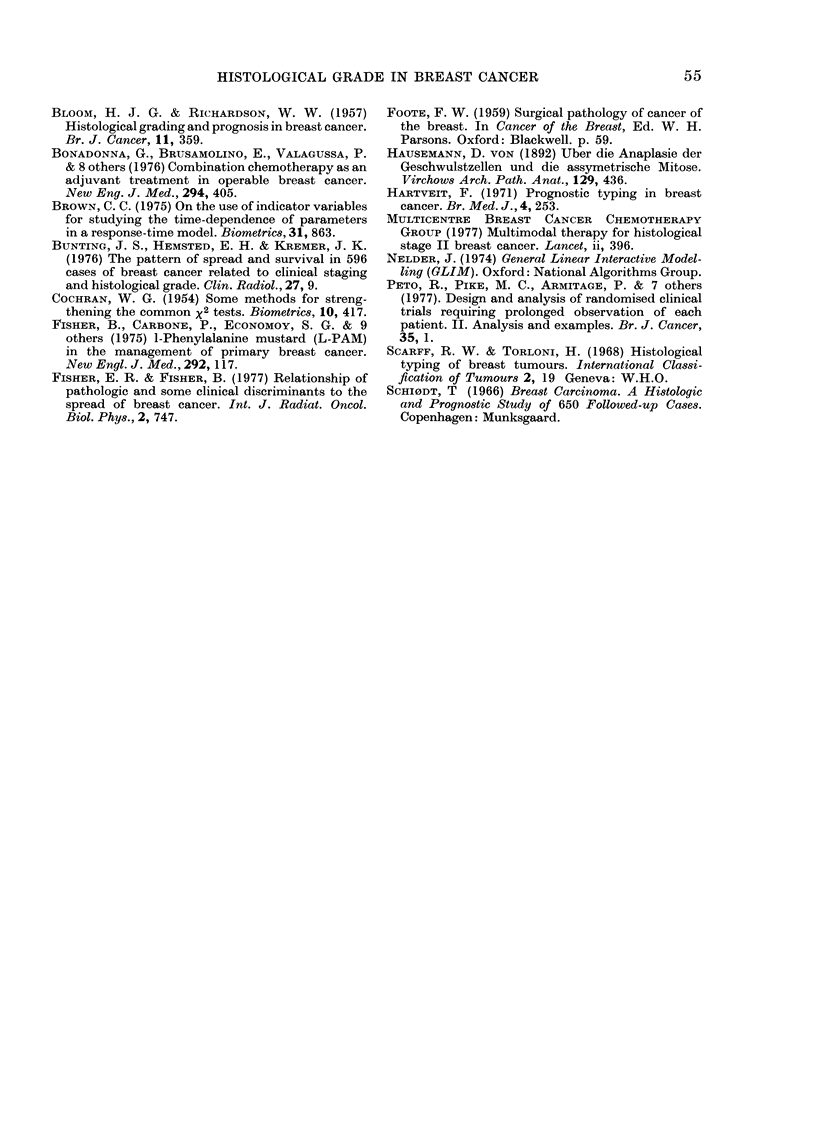# Histological grade and other prognostic factors in relation to survival of patients with breast cancer.

**DOI:** 10.1038/bjc.1979.139

**Published:** 1979-07

**Authors:** L. S. Freedman, D. N. Edwards, E. M. McConnell, D. Y. Downham

## Abstract

**Images:**


*Br. J. Cancer* (1979) **40**, 44

### HISTOLOGICAL GRADE AND OTHER PROGNOSTIC FACTORS IN RELATION TO SURVIVAL OF PATIENTS WITH BREAST CANCER

L. S. FREEDMAN*, D. N. EDWARDS*, E. M. McCONNELL†, and D. Y. DOWNHAM‡

*From the *Clinical Research Unit, Mersey Regional Centre for Radiotherapy and Oncology, the †Histopathology Register, Mersey Regional Cancer Registry, Clatterbridge Hospital, Bebington, Merseyside, and the ‡Department of Computational Science and Statistics, University of Liverpool*

Received 24 April 1978 Accepted 12 March 1979

**Summary.—Records of 3085 patients registered with breast cancer at the Mersey Regional Cancer Registry have been analysed to evaluate the relative importance of possible prognostic factors. In a subgroup of 1759 patients, clinical stage and histological grade are shown to be strongly related to survival after treatment. In addition histological grade is related to the *distribution* of times to death after treatment. The results of this and 3 other studies have implications for the design and analysis of clinical trials in the primary treatment of breast cancer.**

SINCE the introduction of histological grading in breast cancer by von Hausemann (1892) its contribution to knowledge of the disease and its appropriate role in the choice of treatment has been a matter of dispute. A primary difficulty with most grading methods has been the lack of reproducibility in the assessment of grade, both by different pathologists and by the same pathologist on different occasions (Foote, 1959). Not unreasonably these difficulties have led to some resistance to the wide adoption of one of these methods for the purpose of guidance in the management of treatment.

Nevertheless Bloom (1950; 1962) and Bloom & Richardson (1957) showed how successful grading by one pathologist could be in predicting survival. Schiødt (1966) reviewed many of the different grading methods proposed, and found Bloom & Richardson's (1957) method conveniently simple to apply. Both Schiødt and Bloom found the reproducibility of grade on repeated assessments by the same pathologist to be acceptably high. This method of grading has since been adopted by WHO (Scarff & Torloni, 1968). Schiødt (1966) and Bunting *et al.* (1976) have both confirmed the close relationship of grade to length of survival following treatment. Hartveit (1971) has discussed another grading method for which she claims a greater predictive power than Bloom's method, but her results are as yet unconfirmed.

Since 1958 one of us (E.M.McC.) has been grading breast carcinomas according to Bloom and Richardson's method, using histological material submitted to the Mersey Regional Cancer Registry by pathologists in the Liverpool region, North Wales and the Isle of Man. This paper examines the survival of patients treated by radical surgery in relation to the histological grade and other prognostic factors. Recently statistical methodology has been developed to allow simultaneous assessment of several prognostic factors in survival. One such method (Brown, 1975) has been used in this investigation.

Correspondence to: L. S. Freedman, MRC Cancer Trials Office, The Medical School, Hills Road, Cambridge CB2 2QH.

*Data*

The study is based on the records of the Mersey Regional Cancer Registry (MRCR) for the years 1961–68, which registers from a population of an area comprising the Liverpool Regional Hospital Board, the 5 old counties of North Wales (Anglesey, Caernarvonshire, Denbighshire, Flintshire, and Merionethshire) and the Isle of Man. During this period a total of 7493 cases of carcinoma of the breast were registered, but this study is limited to 3085 patients who were initially treated by radical surgery only. Of these cases, histological material in the form of a duplicate section was available at the Cancer Registry for 1759 patients classified as having infiltrating carcinoma. Scarff & Torloni (1968) recommend the term "infiltrating carcinoma" to be used to include all carcinomas of the breast except intraduct and intralobular non-infiltrating carcinomas, and the following general histological variants of invasive carcinoma: medullary carcinoma, papillary carcinoma, cribiform carcinoma, mucous carcinoma, lobular carcinoma, squamous-cell carcinoma and Paget's disease of the breast. Thus the group of infiltrating carcinoma contains a large range of malignant epithelial growths, from well-differentiated adenocarcinoma to completely undifferentiated carcinoma.

The grading system was that recommended by Bloom & Richardson and took account of the following histological criteria:

(1) Tubule formation
(2) Hyperchromatism and mitosis
(3) Irregularity of size, shape and staining of nuclei.

Well-marked tubule formation is characteristic of a high degree of differentiation and indicates a favourable prognosis, and 1 point is awarded if the section shows well-marked tubule formation, 2 points if tubule formation is moderate and 3 points if there is little or no differentiation.

The greater the number of hyperchromatic or mitotic nuclei the worse the prognosis. One point is awarded if only an occasional hyperchromatic nucleus or mitotic figure is seen per high-power field, 2 points if there are 2–3 such figures in most fields and 3 points if the number is higher. One point is awarded if the nuclei are uniform in size, shape and staining, 2 points if there is moderate variation and 3 points if pleomorphism is marked.

The points allocated for each of the 3 criteria are added together; a total of 3–5 is graded I, 6–7 points graded II, and 8–9 points is graded III.

In most cases one section was sufficient for grading, but with larger growths it was sometimes necessary to examine several sections. When there was a definite variation in grade within a tumour then the grading of the most malignant area was allotted.

TABLE I.—*Items used in the analyses and the selected groups*

| Item of information | Groups |
|---|---|
| Prognostic factors— | |
| Age of patient: | <45 yrs; 45–54 yrs; 55 yrs plus; |
| Size of tumour at presentation: | Unknown; <5 cm; 5 $cm^{+}$ |
| Clinical stage of disease: | Grade I; Grade II; Grade III; unknown |
| Histological grade of tumour: | Stage 1; Stage 2; Stage 3. |
| Treatment— | |
| Type of initial treatment (radical): | Surgery |
| Year of initial treatment: | 1961–1968 inclusive |
| Response— | |
| Length of survival*: | Each completed year of survival |
| Survival status: | Alive; Dead, cause unknown; Dead, no malignancy; Dead, malignancy; Dead, date unknown; Not known whether dead or alive |

*Measured from date of initial treatment.

The items of information, in the coded form on magnetic tape which is used in this study, are shown on Table I. All these items were checked with the more detailed registry case sheets and found to be consistent. However, certain of the items are open to errors which are inherent in any retrospective survey.

For example, the clinical assessment of the size of the tumour was copied from the hospital notes whenever it was recorded, but frequently the clinician had failed to

FIG. 1.—Photomicrograph of a Grade 1 carcinoma (H and E. ×60)

FIG. 2.—Photomicrograph of a Grade 2 carcinoma (H and E. ×170).

FIG. 3.—Photomicrograph of a Grade 3 carcinoma (H and E. ×170).

record this factor. It is recognised by most clinicians that the measurement cannot be very accurate. Clinical staging was assessed using the "Manchester" system. The stage was usually decided by non-medical staff of the registry on reading the hospital notes and there is some evidence to show that this is more accurate than staging done infrequently by junior doctors. The staff had been trained for this work and probably the allotted stage can be accepted as the best information available. Histological grade had been decided by the one pathologist throughout (E.M.McC.), at the time of the initial registration. The grade, of course, was allotted only if a histological section had been submitted to the Registry. Photomicrographs of breast tumours typical of the grade given are shown (Fig. 1, 2 and 3). An analysis of the early years of grading (1958–60) showed considerable variation of the proportions of the grades, but for the years of analysis these were more stable (Table II). Disease status at death (presence or absence of malignant disease) is thought to be a reasonably accurate record, being based on clinical assessment summarized in the annual follow-up information on the patient. It should be more

TABLE II.—*Distribution of histological grade by year of diagnosis**

| Year of diagnosis | Histological grade | | | | |
|---|---|---|---|---|---|
| | I† | II | III | Unknown | Total |
| 1961 | 32 (13·1) | 85 (34·8) | 128 (52·5) | 643 | 888 |
| 1962 | 28 (13·6) | 79 (38·3) | 99 (48·1) | 656 | 862 |
| 1963 | 56 (21·5) | 96 (36·9) | 108 (41·5) | 722 | 982 |
| 1964 | 56 (17·7) | 128 (40·4) | 133 (42·0) | 624 | 941 |
| 1965 | 80 (22·0) | 119 (32·7) | 165 (45·3) | 516 | 880 |
| 1966 | 116 (28·4) | 124 (30·4) | 168 (41·2) | 571 | 979 |
| 1967 | 99 (25·1) | 134 (33·9) | 162 (41·0) | 569 | 964 |
| 1968 | 92 (25·4) | 114 (31·5) | 156 (43·1) | 635 | 997 |
| Total | 559 | 879 | 1119 | 4936 | 7493 |

* This table is not restricted to radically treated patients but includes all patients diagnosed 1961–1968.

† % of the total number of tumours allotted a grade during the year are in parentheses.

reliable than the cause of death given on the death certificate.

*Statistical analysis*

The 4 prognostic factors used in this study are listed in Table III. A patient may belong to any one of the possible 108 (=3×3×3×4) prognostic groups. A cross-tabulation of "survival status" against length of survival is obtained for each prognostic group. As a by-product, the cross-tabulations of the prognostic factors themselves are also obtained. The relationship between the various prognostic factors and survival can be assessed by studying appropriate life-tables (Armitage, 1971) which are formed from the cross-tabulations. Two types of life-table are formed: that considering all causes of death together, and that considering deaths from malignant disease only, other deaths being counted as lost to follow-up. Survival curves were drawn to illustrate the information in these life-tables and several are presented in the Results section. These figures and also the tables of survival rates refer to all causes of death.

TABLE III. — *Distribution of prognostic factors in the study*

| | All patients (3085) | Subgroup with sections available to grade (1759) |
|---|---|---|
| Age <45 | 427 (13·8) | 242 (13·8) |
| 45–54 | 782 (25·3) | 445 (25·3) |
| 55 plus | 1876 (60·8) | 1072 (60·9) |
| Tumour size <5 cm | 309 (10·0) | 212 (12·1) |
| 5 cm plus | 598 (19·4) | 368 (20·9) |
| unknown | 2178 (70·6) | 1179 (67·0) |
| Clinical stage 1 | 1565 (50·7) | 854 (48·6) |
| 2 | 965 (31·3) | 591 (33·6) |
| 3 | 555 (18·0) | 314 (17·9) |
| Histological grade | | |
| I | 384 (12·4) | 384 (21·8) |
| II | 616 (20·0) | 616 (35·0) |
| III | 759 (24·6) | 759 (43·1) |
| unknown | 1326 (43·0) | — (—) |

For a more formal assessment of the relationships between the prognostic factors and survival a mathematical model must be introduced. A model of Brown (1975) seems appropriate for our data and a description is given in Appendix I. The analyses of the data using this model are performed by a programming package known as GLIM, that is available on the Liverpool University computer. The input for the programme consists of extracts from the 108 cross-tabulations mentioned earlier in this section. Each analysis (all causes of death and deaths from malignant disease) was performed on all 3085 records and repeated for the subset of 1759 records for which a histological grade had been allocated. The results of the analysis of deaths from all causes are described in some detail in this paper, and the results of death from malignant disease are described briefly in Appendix 2.

### RESULTS

Table III shows the distribution of each prognostic factor among the 3085 patients, and also among the subgroup of 1759 patients allocated a tumour grade. Two points that might be stressed are that the size of tumour was recorded in only 30% of the records and that the distribution of the 1759 patients over histopathological grades I, II, and III was in the approximate 3:5:6 ratio.

Table IV shows the 5-year survival rates (all causes of death considered) estimated from life-tables for each prognostic factor. Since the most important factors appear from this table to be clinical stage and histological grade, the life-tables for each *combination* of clinical stage and histological grade were also computed. Figs. 4, 5 and 6 show the survival curves plotted from these life-tables on a logarithmic scale.

The results of the analysis using the mathematical model (Brown, 1975) are presented in detail in Appendix 2. These results indicate that, of the 4 prognostic factors considered, only histological grade and clinical stage are in fact of importance; moreover in this series histological grade is found to be more important than

TABLE IV.—*Survival (%) at 5 and 10 years by prognostic factor*

| | All records (3085) | | Subgroup with grade (1759) | |
|---|---|---|---|---|
| | 5 yr | 10 yr | 5 yr | 10 yr |
| Age <45 | 67·7 | 51·9 | 69·0 | 52·8 |
| 45–54 | 63·5 | 47·4 | 65·9 | 48·3 |
| 55 plus | 57·7 | 33·8 | 57·9 | 34·4 |
| Tumour size unknown | 59·3 | 38·8 | 60·6 | 39·0 |
| <5 cm | 64·1 | 41·8 | 63·2 | 43·4 |
| 5 cm plus | 62·0 | 41·5 | 62·7 | 43·6 |
| Clinical Stage 1 | 67·6 | 47·4 | 68·1 | 47·8 |
| 2 | 55·7 | 34·7 | 57·6 | 36·0 |
| 3 | 47·9 | 26·5 | 49·7 | 28·8 |
| Histological Grade I | 77·9 | 56·3 | 77·9 | 56·3 |
| II | 64·2 | 39·4 | 64·2 | 39·4 |
| III | 50·6 | 33·3 | 50·6 | 33·3 |
| unknown | 59·0 | 38·6 | — | — |

FIG. 4.—Survival curves. Histological Grade I —— Stage 1; – – – Stage 2; ..... Stage 3.

FIG. 5.—Survival curves. Histological Grade II. —— Stage 1; – – – Stage 2; ..... Stage 3.

clinical stage. In addition the analysis shows that the *distribution* of deaths over the period after treatment varies according to the histological grade but not according to the clinical stage. This phenomenon is illustrated by plotting the "risk of death" against time for the 3 histological grades and 3 clinical stages. (The "risk of death" is defined as the number of deaths during one year expressed as a proportion of the numbers of persons at risk during that year.) The plots are shown in Fig. 7 and 8. In Fig. 7 it can be seen that the peak risk for patients with Grade III tumours is reached at an earlier period (between the first and second year after treatment) than for those with lower grade tumours. In

FIG. 6.—Survival Curves. Histological Grade III. —— Stage 1; – – – Stage 2; ..... Stage 3.

FIG. 7.—"Risks of Death" for 3 Histological Grades. —— Grade I; – – – Grade II; ..... Grade III.

FIG. 8.—"Risks of Death" for 3 Clinical Stages. —— Stage 1; – – – Stage 2; ..... Stage 3.

contrast, Fig. 8 shows no such difference between the groups of patients with different clinical stages of disease.

### DISCUSSION

The results of this retrospective analysis suggest the importance of histopathological grade as a prognostic factor in breast cancer. Our results are in basic agreement with those of Bloom & Richardson (1957), Schiødt (1966) and Bunting *et al.* (1976), but differences in some of the details need to be discussed. The distribution over Grades I, II and III in our series was in the ratio 3:5:6, whereas in the 3 reports cited above the ratios are nearer 1:2:1. It seems likely that this difference is due to the subjective nature of grading, and indicates that Bloom's method, although the most clearly documented of available methods (Schiødt, 1966), may be liable to substantial differences in interpretation among pathologists. The alternative explanation of a real difference in the tumours in this geographical region cannot be completely ruled out. Although, over the period of this study (1961–1968) the ratios are more stable than in the earlier years, the ratios at the end of our series can be seen in Table II to be different from those at the beginning. The changes in the ratios are most pronounced at the start of the series, and presumably this coincided with the period in which the pathologist was gaining experience in this grading method. Our analysis, unlike those of Bloom (1962) and Bunting *et al.* (1976) shows histological grade to be more important than clinical stage. However, this is possibly a reflection of the inadequate recording by clinicians of the clinical stage. Despite all these differences, the relationship between histological grade and prognosis holds as strongly in this study as in the 3 previous studies.

Bloom, Bunting and Schiødt all comment on the different time-patterns of deaths according to tumour grade. This is confirmed in our study. For Grade III tumours, the peak rise of death is between the first and second year after treatment, and for Grades I and II tumours the risk of death reaches its peak between 4–6 years after treatment. However, further analysis (not described here) suggests that the position of peaks in these latter two groups may depend on the clinical stage, but a larger number of patients and a longer follow-up would be required to clarify this point.

The importance of Bloom's histological grade as a prognostic factor, as now demonstrated in 4 independent reports involving large numbers of breast-cancer patients, raises issues on the design, analysis and interpretation of clinical trials in the primary management of this disease.

By including histological grade as a covariate in the statistical comparison of two treatments (Armitage & Gehan, 1974) we would make such a comparison more precise. For example, it can be shown that if Bloom's results (1962) hold true in a trial of treatment for Clinical Stage 2 disease, including histological grade in the analysis would be equivalent to having 13% more patients in the trial (see Appendix 3). Thus, by arranging for the tumours to be graded by a pathologist, it should be possible to obtain an answer from the trial at an earlier date. In addition such a trial could tell us whether the difference in response to each treatment was the same for high-grade and low-grade tumours. For example, cytotoxic therapy adjuvant to surgery may be more effective than surgery alone for high-grade tumours but not for low-grade tumours. Fisher & Fisher (1977) have recently made a similar recommendation that histological information be used in breast cancer trials.

It should be noted that if histological grade is used in the analysis of breast-cancer studies based on survival data, comparing the survival according to grade by the logrank test (Peto *et al.*, 1977) is not particularly appropriate, since the "risks of death" (or hazards) for the different grades do not bear constant ratio to each other over time. This point is illustrated in Fig. 7. In such a case time-dependent variables should be introduced, and among others Brown's (1975) method used in this study might be considered. However, it may still be appropriate to compare the treatment groups themselves by the logrank test after the appropriate stratification (Peto *et al.*, 1977).

The data presented here also have implications for the results of trials in breast cancer which have been reported after follow-up times of at most 3 years (Fisher *et al.*, 1975; Bonadonna *et al.*, 1976; Multicentre Breast Cancer Chemotherapy Group, 1977). Differences between treatments found in these studies may reflect only the response of Grade III tumours (Fig. 7). It may be too early to say whether tumours of low grades respond in the same way.

We are grateful to Dr J. Haybittle for his helpful criticisms and to Mr C. West who helped with some of the computing. We also thank Miss Sheila Murray for typing the paper.

### APPENDIX I. APPLICATION OF BROWN'S MODEL

We illustrate the method using the simple case where the relationship between survival and a single prognostic factor is to be assessed. We suppose that the prognostic factor has 2 possible values. The time after the first treatment is divided into a number of periods. In this study 6 periods were chosen: 0–1 year, 1–2 years, 2–4, 4–6, 6–8 and 8–10 years.

To describe Brown's model it is necessary to introduce the idea of a "hazard". The hazard for a particular period is defined as the probability that a patient who has survived to the beginning of the period will die at some time during that period. Thus for a period of one year the hazard is identical to the "risk of death" which was introduced in the Results section of the main text, while for a period of 2 years the hazard is twice the risk of death.

Let the hazard for a patient who has value $i$ of the prognostic factor (where $i=1$, or 2) and is at risk during Period $t$ (where $t=1$, 2, 3, 4, 5, or 6) be denoted by $P_{it}$. Brown's model postulates that

$$\text{logit}\, P_{it}=\log\left(\frac{P_{it}}{1\text{-}P_{it}}\right)=M+V_{\text{i}}+T_{\text{t}}+(VT)_{it} \quad (i=1,\ 2;\ t=1,\ 2,\ 3,\ 4,\ 5,\ 6) \quad \text{(A1)}$$

where $M$ is the overall effect, $V_i$ is an effect due to the $i$th value of the prognostic factor, $T_t$ is an effect due to the $t$th time period and $(VT)_{it}$ is an effect due to the "interaction" between the prognostic factor and the periods.

The values of $M$, $V_i$, $T_t$ and $(VT)_{it}$ can be so chosen that the data used in this study are fitted exactly by the model. However, if certain of the terms on the right-hand side of expression (A1) are constrained to be equal to zero, the data will not necessarily be fitted exactly. The goodness-of-fit of such a constrained model is measured by the value of twice the maximized log-likelihood; whenever this value is significantly large, the corresponding model is rejected as an inadequate description of the data. In our example the constrained models of interest are:

$$\text{logit } P_{it}=M \quad \text{(A2a)}$$

$$\text{logit } P_{it}=M+T_t \quad \text{(A2b)}$$

$$\text{logit } P_{it}=M+V_i+T_t \quad \text{(A2c)}$$

The time-effect $T_t$ is regarded as a basic component of the model, and the prognostic factor effect $V_i$ is therefore never included in the model without $T_t$. The analysis proceeds by searching through these models to find the simplest (*i.e.* the model with the least terms) which is judged an adequate description of the data. If for example model (A2c) is judged to be an adequate description, it follows that the interaction terms $(VT)_{it}$ are not really required.

Another way of viewing the results is to consider how much improvement is achieved in the goodness-of-fit by adding extra terms into the model. For example, if the goodness-of-fit criterion for Model A2c is not much smaller than that for Model A2b, it would follow that the terms $V_i$ add little to the description of the data, once the terms $M$ and $T_t$ have been included. Thus one may measure the *importance* of prognostic factors by the differences in the goodness-of-fit criteria of the 2 appropriately chosen models.

The method can be used in a similar way to examine simultaneously the importance of several prognostic factors. The analysis was carried out using the GLIM programme package (Nelder, 1974) that is available on the main Liverpool University computer, an ICL 1906S. Unfortunately, at the time of analysis there were restrictions on the storage space allowed to GLIM at Liverpool University, and as a result the analysis had to be performed in piecemeal fashion, assessing the prognostic factors three at a time instead of all at once.

As mentioned in the main text, the data were analysed in two parts; firstly including all 3085 records, and secondly the

TABLE A1.—*Results of fitting Brown's model to the data of* 3085 *patients*

| Model§ | Goodness-of-fit criteria | Degrees of freedom | $P$* | Measure of importance† (relevant factor shown in parentheses) |
|---|---|---|---|---|
| M | 618·9 | 71 | <0·0001 | — |
| M+T | 274·0 | 66 | <0·0001 | 59·0 (T) |
| M+T+S | 171·3 | 64 | <0·0001 | 51·3 (S) |
| M+T+G | 193·1 | 63 | <0·0001 | 27·0 (G) |
| M+T+S+G | 96·95 | 61 | <0·0001 | — |
| M+T+S+G+T.S | 82·06 | 51 | 0·004 | 1·5 (T.S.) |
| M+T+S+G+T.G | 42·67 | 46 | 0·59 | 3·6 (T.G.) |
| M+T+S+G+T.S+T.G | 27·46 | 36 | 0·84 | — |

§ A value of $P$ which is less than 0·05 implies that the model does not adequately fit the data.

† Calculated as the reduction in the goodness-of-fit criterion achieved by adding the factor into the model divided by the reduction in the degrees of freedom.

§ M=Overall mean, T=Time period, S=Clinical stage, G=Histological grade.

TABLE A2.—*Results of fitting Brown's model to the data of* 1759 *patients with histological grade*

| Model | Goodness-of-fit | Degrees of freedom | $P$ | Measure of importance (Factor) |
|---|---|---|---|---|
| M | 424·6 | 53 | <0·0001 | — |
| M+T | 190·5 | 48 | <0·0001 | 46·8 (T) |
| M+T+S | 144·6 | 46 | <0·0001 | 22·9 (S) |
| M+T+G | 109·4 | 46 | <0·0001 | 40·5 (G) |
| M+T+S+G | 70·57 | 44 | 0·007 | — |
| M+T+S+G+T.S | 61·33 | 34 | 0·003 | 0·9 (T.S) |
| M+T+S+G+T.G | 25·33 | 34 | 0·86 | 3·6 (T.G) |
| M+T+S+G+T.S+T.G | 16·27 | 24 | 0·88 | — |

Abbreviations as for Table A1.

subset of 1759 records with a histological grade.

### APPENDIX 2.—RESULTS OF ANALYSIS USING BROWN'S MODEL

The factors were assessed in the following combinations:

(i) Time, clinical stage, histological grade

(ii) Time, clinical stage, histological grade, age

(iii) Time, clinical stage, histological grade, tumour size.

The first combination was chosen because it was clear from published work and from a preliminary scan of our data that time, stage and grade would have to be included in our model. Each of the other prognostic factors were then added to determine whether it made any further contribution to the prediction of survival. This approach is not normally recommended but in this study we were forced into it by the restrictions in the use of GLIM on the Liverpool computer.

In the analysis of each combination of factors the models were considered in a sequence similar to the models A2a–A2c. The goodness-of-fit criterion was obtained for each model examined. The results of the analysis of Combination (i) are shown in Tables A1 and A2, which refer to the analysis of all 3085 patients and the smaller group of patients with histological grades respectively. These tables show the goodness-of-fit criteria with their corresponding degrees of freedom and probability levels. The latter are calculated assuming that the criterion has a chi-squared distribution with the given degrees of freedom. The final column gives a value (based on the difference between the goodness-of-fit criteria of two models) which measures the importance of a factor or an interaction between two factors.

Tables A1 and A2 show that, as well as the main effects of time, stage and grade, the interaction of time and grade is required in the model. It was the latter result which led to our plotting Fig. 4 and 5 of the main text. The order of importance of stage and grade is reversed in the two tables. This was because the analysis of Table A1 included an extra histological grade, classified as unknown, and this inevitably "muddied" the contribution played by grade. The comparison of the importance of stage and grade in Table A2 is therefore the more reliable (but see the Discussion section in the main text for further comments).

The prognostic factor tumour size was not found to be of any importance, once grade and stage were included in the model. Age, however, was of importance, both as a main effect and also through an age–grade interaction. It was found that on further analysis, when deaths from malignant disease only were considered, the contribution of age became negligible, although time, stage, grade and the time–grade interaction remained important.

We concluded therefore that the age effect was due to deaths from non-malignant disease in old age. In the interests of brevity the results of these analyses are not included.

### APPENDIX 3.—EFFECT OF INCLUDING HISTOLOGICAL GRADE IN ANALYSING THE RESULTS OF A CLINICAL TRIAL

Suppose we enter $2N$ patients with Clinical Stage 2 disease into our trial. Let $N$ patients receive the conventional treatment (A) and $N$ receive the alternative treatment (B). Suppose also that in the total group of $2N$ patients, the number with histological grades I, II and III are 0·5N, $N$ and $0{\cdot}5N$ respectively. Finally, suppose that experience shows that the 5-year survival rate for patients with Grades I, II and III following conventional therapy (A) are 71%, 43% and 24% respectively. These figures are taken from Bloom (1962).

We will consider a comparison of Treatments A and B based on 5-year survival rates. Normally one would use the survival times themselves in any treatment comparison (Peto *et al.*, 1977). However, we use here the cruder method of comparing the 5-year rates for the sake of simplicity.

Assume firstly that no knowledge of histological grade has been obtained for this trial. Then the expected 5-year survival rate in the treatment Group A is:

$$(0{\cdot}25\times 71\%)+(0{\cdot}5\times 43\%)+(0{\cdot}25\times 24\%)=45{\cdot}25\%$$

Under the null hypothesis (that there is no difference between treatments) the standard error of the difference in proportions is:

$$\sqrt{\frac{2\times 45{\cdot}25\%\times 54{\cdot}75\%}{N}}=\frac{70{\cdot}4\%}{\sqrt{N}}$$

Now assume that the histological grade is known for each patient in the trial. Let the numbers of Grade Is, IIs and IIIs be $n_1$, $n_2$, $n_3$, in treatment Group A, and $m_1$, $m_2$, $m_3$ in treatment Group B. We may now use Cochran's (1954) test to compare the treatments. We compute $\bar{d}$, given by:

$$\bar{d}=\sum_{i=1}^{3} w_i d_i \Big/ \sum_{i=1}^{3} w_i$$

where $d_i$ is the difference in survival rates of the two treatment groups for Histological Grade $i$ ($i=1$, 2, 3) and

$$w_i=\frac{n_i m_i}{n_i+m_i}$$

Suppose that an equal distribution of grades has in fact taken place over the two treatment groups, *i.e.* $n_1=m_1$, $n_2=m_2$, $n_3=m_3$ (small departures from this assumption do not alter the validity of the argument). Then:

$$w_i=n_i/2,\ n_1=0{\cdot}25N,\ n_2=0{\cdot}5N \text{ and } n_3=0{\cdot}25N$$

and the expression for $\bar{d}$ reduces simply to the difference in the 5-year survival rates of the two groups as before. However, the standard error of $\bar{d}$ now becomes equal to:

$$\sqrt{2[(0{\cdot}25\times 71\%\times 29\%)+(0{\cdot}5\times 43\%\times 57\%)+(0{\cdot}25\times 24\%\times 76\%)]/N}=\frac{66{\cdot}3\%}{\sqrt{N}}$$

Therefore $N$ patients and a knowledge of their histological grade gives as much accuracy in the comparison as $N'$ patients without a knowledge of their grade where

$$\frac{66{\cdot}3}{\sqrt{N}}=\frac{70{\cdot}4}{\sqrt{N'}}\ \textit{i.e.}\text{ where } N'=1{\cdot}13\,N$$

Thus we have the equivalent of 13% more patients in the trial.

### REFERENCES

Armitage, P. (1971) *Statistical Methods in Medical Research*, Ch. 14, Oxford: Blackwell.

Armitage, P. & Gehan, E. A. (1974) Statistical methods for the identification and use of prognostic factors. *Int. J. Cancer*, **13**, 16.

Bloom, H. J. G. (1950) Prognosis in carcinoma of the breast. *Br. J. Cancer*, **4**, 259.

Bloom, H. J. G. (1962) The role of histological grading in the study of breast cancer. In *Symposium on the Prognosis of Malignant Tumours of the Breast* Ed. P. Denoix & C. Rouquette. *UICC Acta*, **18**, 842.

BLOOM, H. J. G. & RICHARDSON, W. W. (1957) Histological grading and prognosis in breast cancer. *Br. J. Cancer*, **11**, 359.

BONADONNA, G., BRUSAMOLINO, E., VALAGUSSA, P. & 8 others (1976) Combination chemotherapy as an adjuvant treatment in operable breast cancer. *New Eng. J. Med.*, **294**, 405.

BROWN, C. C. (1975) On the use of indicator variables for studying the time-dependence of parameters in a response-time model. *Biometrics*, **31**, 863.

BUNTING, J. S., HEMSTED, E. H. & KREMER, J. K. (1976) The pattern of spread and survival in 596 cases of breast cancer related to clinical staging and histological grade. *Clin. Radiol.*, **27**, 9.

COCHRAN, W. G. (1954) Some methods for strengthening the common $\chi^2$ tests. *Biometrics*, **10**, 417.

FISHER, B., CARBONE, P., ECONOMOY, S. G. & 9 others (1975) l-Phenylalanine mustard (L-PAM) in the management of primary breast cancer. *New Engl. J. Med.*, **292**, 117.

FISHER, E. R. & FISHER, B. (1977) Relationship of pathologic and some clinical discriminants to the spread of breast cancer. *Int. J. Radiat. Oncol. Biol. Phys.*, **2**, 747.

FOOTE, F. W. (1959) Surgical pathology of cancer of the breast. In *Cancer of the Breast*, Ed. W. H. Parsons. Oxford: Blackwell. p. 59.

HAUSEMANN, D. VON (1892) Uber die Anaplasie der Geschwulstzellen und die assymetrische Mitose. *Virchows Arch. Path. Anat.*, **129**, 436.

HARTVEIT, F. (1971) Prognostic typing in breast cancer. *Br. Med. J.*, **4**, 253.

MULTICENTRE BREAST CANCER CHEMOTHERAPY GROUP (1977) Multimodal therapy for histological stage II breast cancer. *Lancet*, ii, 396.

NELDER, J. (1974) *General Linear Interactive Modelling (GLIM)*. Oxford: National Algorithms Group.

PETO, R., PIKE, M. C., ARMITAGE, P. & 7 others (1977). Design and analysis of randomised clinical trials requiring prolonged observation of each patient. II. Analysis and examples. *Br. J. Cancer*, **35**, 1.

SCARFF, R. W. & TORLONI, H. (1968) Histological typing of breast tumours. *International Classification of Tumours* **2**, 19 Geneva: W.H.O.

SCHIØDT, T (1966) *Breast Carcinoma. A Histologic and Prognostic Study of 650 Followed-up Cases.* Copenhagen: Munksgaard.